# Synergistic Leaching of Titanium, Aluminum, and Magnesium Components during Dilute Acid Pressure Treatment of High-Titanium Blast Furnace Slag

**DOI:** 10.3390/molecules29143336

**Published:** 2024-07-16

**Authors:** Ke Yuan, Siqi He, Bo Yu, Shiyi Qian, Xueyu Wu, Wenyi Li, Chunmeng Zhao

**Affiliations:** School of Resources & Environment Engineering, Mianyang Teachers’ College, Mianyang 621000, China

**Keywords:** high-titanium blast furnace slag, dilute sulfuric acid, pressure, acid digestion, filtration

## Abstract

This study focuses on an improved leaching process through the combination of pressurized conditions and direct filtration of acid leaching slurry, which is conductive to improving the filterability of acid leaching systems and the extraction rates of Ti, Al, and Mg components. The effects of sulfuric acid concentration, reaction temperature, particle size of materials, acid–slag ratio, and reaction time on the leaching efficiency were systematically investigated. The results showed that pressurization significantly enhances the filtration efficiency of the reaction slurry. Under the same filtration time, the filtration efficiency increased from 46% under ordinary pressure to 78% under pressurized conditions. Moreover, under the optimal reaction conditions, the extraction rates of Ti, Al, and Mg components were more than 88.21%, 97.8%, and 96.31%, respectively. Additionally, XRD and FTIR showed that titanium oxide sulfate hydrate crystals were produced in the acid-leached residues when the reaction temperature exceeded 190 °C, thereby reducing the extraction rate of Ti component. And the XRD pattern shows that when the reaction temperature is maintained at 190 °C and the reaction time is extended to 150 min, titanium oxide sulfate hydrate crystals will be formed to reduce the extraction rate of the Ti component. In summary, this study not only provides important theoretical support for the resource utilization of high-titanium blast furnace slag but also offers a feasible solution for efficient extraction and convenient filtration, thus holding significant academic and practical implications.

## 1. Introduction

High-titanium blast furnace slag (TBFS) is a kind of slag produced during the smelting of iron using vanadium–titanium magnetite ore, including various compositions, such as perovskite (CaTiO_3_), diopside (CaMg(SiO_3_)_2_), and magnesium aluminum spinel (MgAl_2_O_4_) [1]. The global annual output of TBFS is completely large because of the wide applications of iron. Currently, TBFS are produced mainly in China, Canada, and South Africa [2]. Specifically, the accumulated quantity of TBFS in the Panxi region of China has reached 100 million tons and continues to increase at a rate of 7 million tons per year [3]. TBFS is mainly rich in valuable components such as TiO_2_, Al_2_O_3_, and MgO, so it is a valuable secondary resource [4]. Currently, steel plants dispose of this slag by landfilling, which not only leads to significant waste of valuable components but also poses a series of environmental problems including air pollution, water pollution, and soil contamination [5,6]. Therefore, achieving the resource utilization of TBFS is an unavoidable path towards promoting the green and sustainable development of the mineral industry.

Currently, there are two main methods to utilize the valuable components of TBFS. One is to use TBFS as a whole material without considering the recovery of valuable components. This comprehensive utilization method includes preparing building materials such as sand, ceramics, microcrystalline glass, bricks, and some special functional materials like photocatalysts and composite fertilizers [7,8,9,10,11,12]. The other methods are extracting valuable components such as TiO_2_, Al_2_O_3_, and MgO from TBFS, which is the current mainstream method. Currently, direct extraction methods for these valuable components include the carbonization–chlorination process, synthesis of titanium alloys, acid leaching, and alkali molten salt calcining [13,14,15,16]. In addition, it also includes some unconventional metallurgical enhanced titanium extraction processes, such as microwave metallurgy, super gravity metallurgy, and electromagnetic metallurgy [17,18,19]. Among these methods, acid leaching is also an important method for titanium extraction. The acid leaching method can be conducted at relatively low temperatures to simultaneously reduce energy consumption and efficiently extract different valuable components from TBFS. In addition, the process is simple in operation. However, calcium sulfate colloid is easily produced, which affects the further extraction of titanium and the filtration of the titanium sulfate solution [20]. In order to solve this problem, many researchers have conducted a series of improvement studies. For example, researcher Jiang Tao et al. used sulfuric acid leaching water-quenched TBFS under conditions of a liquid–solid ratio of 10, sulfuric acid concentration of 50%, and temperature of 100 °C, achieving a titanium component extraction rate of 72%. Research results showed that colloidal gypsum easily formed during the sulfuric acid leaching reaction, which adhered to the surface of TBFS and affected a further component leach-out, and the colloidal gypsum also impacted the subsequent liquid–solid phase filtration separation [21]. The research team of Yan Fang used water-quenched TBFS as raw material and extraction Ti, Al, and Mg components use sulfuric acid in two stages. The results showed that under the conditions of 20% sulfuric acid in the first stage, the extraction rates of Ti, Al, and Mg components were 46%, 100%, and 64%, respectively. In the second stage, they applied a 50% sulfuric acid solution to leaching TBFS, simultaneous grinding and leaching, and ultimately achieving a titanium component extraction rate of over 94%. Although this method effectively solved the problem of the colloidal gypsum hindering components extraction rate, it did not significantly improve the filtration performance of reaction slurry [22]. The research team of He Siqi used concentrated sulfuric acid to calcined TBFS and then applied dilute sulphuric acid to the leached reaction product. The results showed that the extraction rates of Ti, Al, and Mg were all above 85%, 96%, and 97%, respectively, and complete liquid–solid separation was achieved within 20 s. Although this method effectively solved the dual issues of a poor extraction rate and poor filtration, in practical operation, serious pollution issues arose due to the use of concentrated sulfuric acid. Furthermore, the method requires two acid treatment stages, making the process relatively complex and unsuitable for industrial applications [20].

In order to solve the above problems, the authors have devised an improved method according to the combination of pressurized conditions and direct filtration of the acid-leached slurry. Firstly, the filterability of acid-leached slurry under ordinary and pressurized conditions was compared. Additionally, the effects of single factor conditions such as sulfuric acid concentration, reaction temperature, particle size of TBFS, acid–slag ratio, and reaction time on the extraction rate of components were systematically discussed. The method of leaching TBFS by sulfuric acid under a pressurized condition accelerates the reaction process and reduces the leaching time. At the same time, this method can improve the extraction rates of valuable components and the filtration efficiency of the acid-leached slurry. In addition, it simplifies the process of treating TBFS with sulfuric acid by reducing the traditional water-leaching procedure, thereby providing a feasible technological approach for large-scale industrial applications.

## 2. Results and Discussions

### 2.1. Effect of Pressure on Filtration Performance of Acid-Leached Slurry

**Figure 1:**
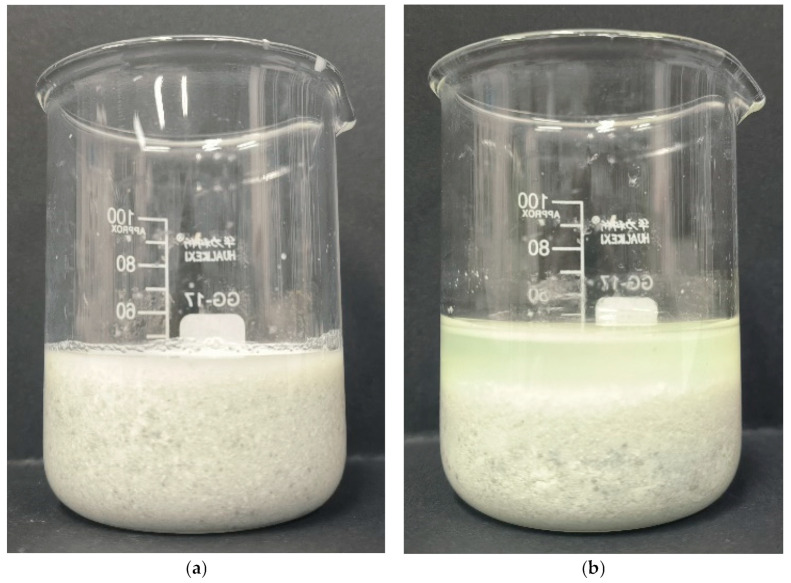
Comparison of acid-leached slurry under ordinary pressure conditions (**a**) and pressurized conditions (**b**) (ωH_2_SO_4_: 30%, acid–slag: 10 mL/g, temperature: 110 °C, time: 4 h).

**Figure 2:**
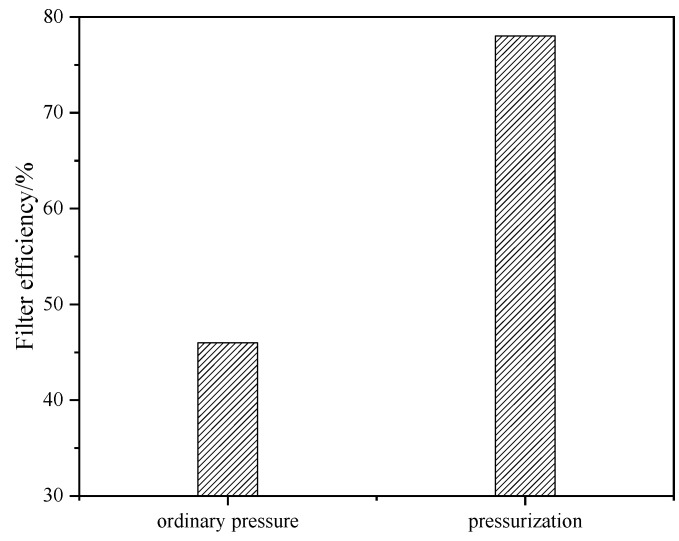
The filter efficiency of acid-leached slurry under two different pressure conditions.

**Figure 3:**
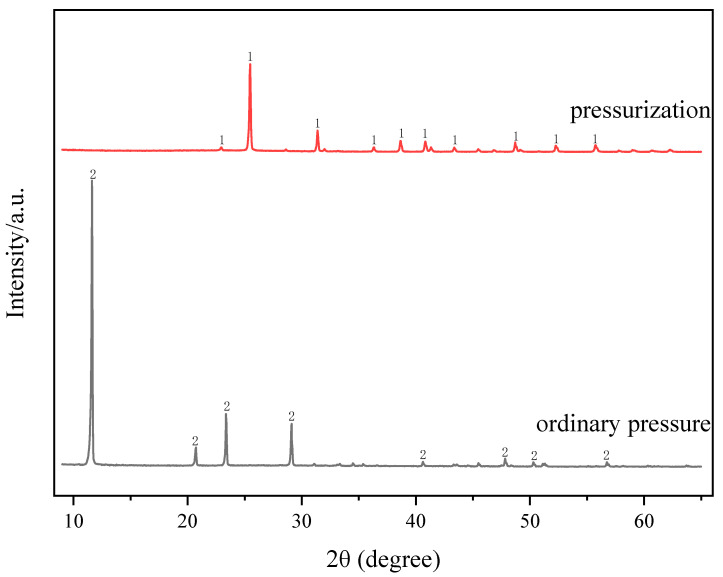
XRD of acid-leached residues under ordinary pressure and pressurized conditions. 1—anhydrite (CaSO_4_) 2—gypsum (CaSO_4_·2H_2_O).

The comparison of filtration performance between ordinary pressure conditions and pressurized conditions is shown in Figure 1 and Figure 2. It can be seen from Figure 1 that there are different reaction phenomena of the acid-leached slurry under different conditions. Under ordinary pressure, the acid-leached slurry exhibits a gelatinous and viscous state, whereas under pressurized conditions, a clear solid–liquid stratified state can be seen. Figure 2 shows that the filtration efficiency of pressurized conditions is 78% and significantly higher than that of ordinary pressure conditions (48%) under the same filtration time (10 min). Previous studies have shown that the solid phase of acid-leached residue from sulfuric acid leaching of TBFS is gypsum or anhydrite. When gypsum is the main reaction product, it can hinder the solid–liquid separation of the acid-leached slurry due to its platy structure. During filtration, these plate-like particles stack horizontally, forming a compact structure that impedes liquid drainage. In contrast, anhydrite facilitates solid–liquid separation because its internal structure consists of numerous thin, vertical “stalactite-like” structures with many gaps between them, conducive to liquid flow [20]. In Figure 3, comparisons of XRD patterns of acid-leached residues under the two pressure conditions show that the main mineral phase in solid product obtained under ordinary pressure conditions and pressurized conditions are gypsum and anhydrite. Studies have shown that if gypsum is placed in a sulfuric acid system, the phenomenon of gypsum (CaSO_4_·2H_2_O) gradually dehydrating into anhydrite (CaSO_4_) will occur, indicating that sulfuric acid plays a role in promoting the transformation of gypsum into II-anhydrite. On the contrary, sulfuric acid should inhibit the reverse transformation of this process [23]. Under the reaction conditions of this design, with the extension of the reaction time, the remaining sulfuric acid in the solution is continuously consumed, and when it is reduced to a certain extent, it cannot provide enough of a sulfuric acid environment. Under ordinary pressure conditions, due to the lack of sulfuric acid environment, the generated gypsum will not remove water molecules to form anhydrite, thus reducing the filtration efficiency. In the closed reactor, due to providing a certain pressure environment, the generated gypsum will remove water molecules to form anhydrite, which is conducive to the filtration of the slurry. Therefore, applying pressure can change the morphology of solid reaction product, so as to improve the filtration efficiency of acid-leached slurry.

The mechanism of gypsum-anhydrite phase transition is analyzed as follows. J·H·Van’t Hoff et al. [24] showed the relationship between the saturated vapor pressure ($P_{2,1/2}$) of diaqueous, an anhydrous calcium sulfate and aqueous system, and the saturated vapor pressure ($P_{W}$) and temperature (T) of pure water at the same temperature (1):

(1) $$\log P_{2,1/2}=\log P_{W}+1.493-567.7/T$$

The equation shows that diaqueous calcium sulfate will gradually transform into anhydrous calcium sulfate when the pressure of the reaction system is equal to the critical pressure at a certain temperature. Under pressurized conditions, the steam pressure in the system can more easily reach the critical condition of the transformation of diaqueous calcium sulfate to anhydrous calcium sulfate than that under ordinary pressure, and finally, all the diaqueous calcium sulfate phase in the pressurized system is transformed into an anhydrous calcium sulfate phase, thus greatly improving the filtration efficiency of the acid-leached slurry in the pressurized system. In addition, the critical condition of conversion from diaqueous calcium sulfate to anhydrous calcium sulfate cannot be reached under ordinary pressure, so the acid leaching residue is mainly calcium sulfate dihydrate, which affects the filterability of the acid leaching system.

### 2.2. Effect of Sulfuric Acid Concentration on Ti, Al, and Mg Extraction Efficiency

**Figure 4:**
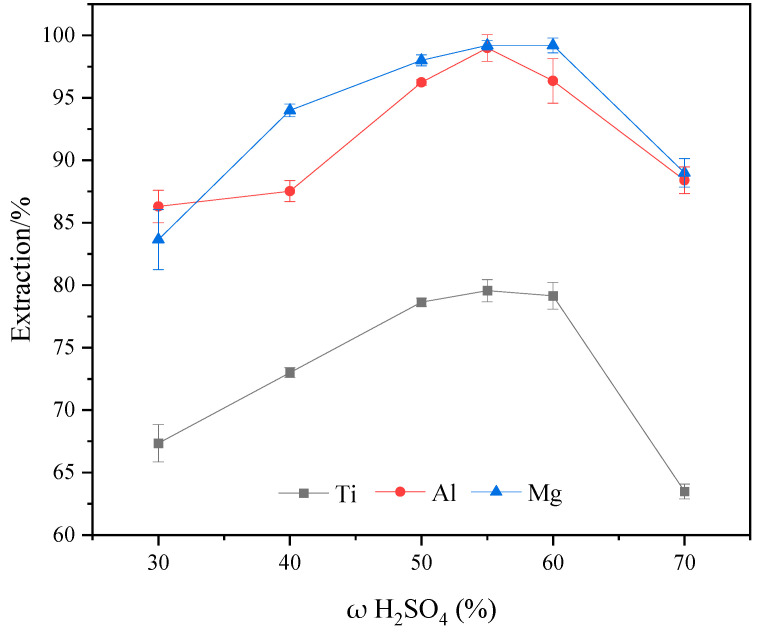
Effect of different sulfuric acid concentrations on the extraction rates of Ti, Al, and Mg (acid–slag: 10 mL/g, temperature: 145 °C, time: 2 h, particle Size: 100–180 M).

**Figure 5:**
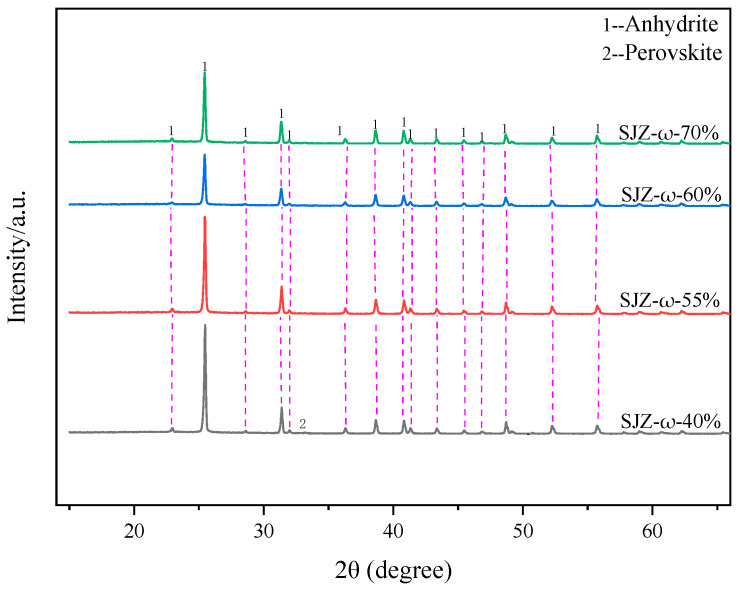
XRD patterns of the acid-leached residues under different sulfuric acid concentrations.

Figure 4 shows the variation in extraction rates of Ti, Al, and Mg components under different sulfuric acid concentrations. It can be observed that when the sulfuric acid concentration is below 55%, the extraction rates of Ti, Al, and Mg increased with the increase in sulfuric acid concentration. When the sulfuric acid concentration is 55%, the extraction rates of Ti, Al, and Mg components reach their maximum values are 79.57%, 98.99%, and 99.21%, respectively. However, when the sulfuric acid concentration is more than 55%, the extraction rates of Ti, Al, and Mg components show a decreasing trend. Figure 5 shows the XRD patterns of the acid-leached residues under different sulfuric acid concentrations. It can be seen from the figure that the main products of the acid-leached residues are anhydrite and a small amount of unreacted perovskite at the sulfuric acid concentration is 40%. As the sulfuric acid concentration is greater than 55%, the main product of the acid-leached residues is anhydrite. The reason for the variation in extraction rates is the reaction between the sulfuric acid and TBFS is not sufficient due to the low-sulfuric acid concentration. Additionally, at lower acid concentrations, hydrolysis of Ti occurs during the leaching process, leading to lower Ti extraction rates [25]. As the sulfuric acid concentration increases, the reaction becomes more complete, resulting in higher component extraction rates. However, when the sulfuric acid concentration is 70%, severe agglomeration between reactants impedes effective reaction, leading to decreased extraction rates of Ti, Al, and Mg components. In summary, the optimal sulfuric acid concentration for acid leaching should be 55%.

### 2.3. Effect of Reaction Temperature on the Extraction Rate of Ti, Al, and Mg

**Figure 6:**
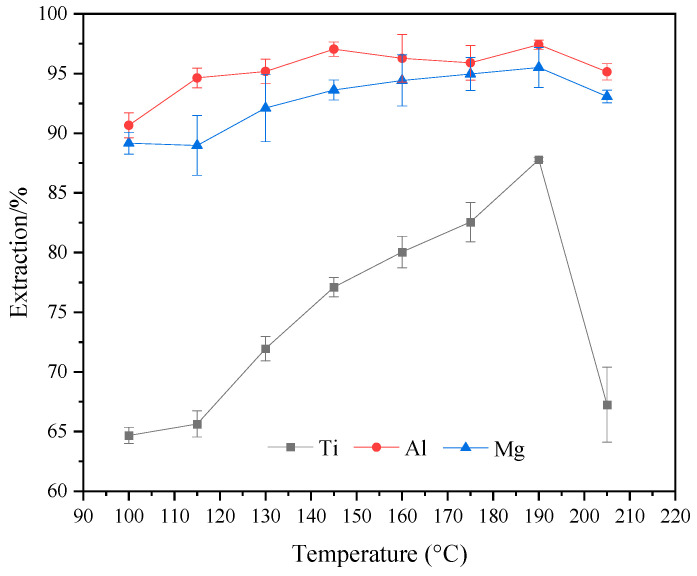
Effect of different reaction temperatures on the extraction rates of Ti, Al, and Mg (ωH_2_SO_4_: 55%, acid–slag: 10 mL/g, time: 2 h, particle size: 100–180 M).

**Figure 7:**
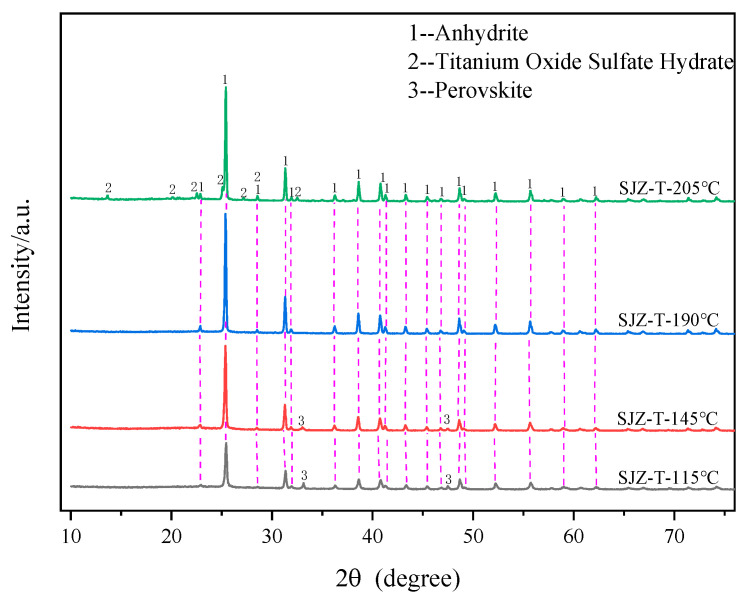
XRD patterns of the acid-leached residues at different reaction temperatures.

Figure 6 shows the variation in extraction rates of Ti, Al, and Mg components at different reaction temperatures. It can be observed from the figure that as the reaction temperature increases, the extraction rate of Ti gradually improves, while the extraction rates of Al and Mg remain above 90%. When the reaction temperature reaches 190 °C, the extraction rates of Ti, Al, and Mg components reach their maximum values of 87.79%, 97.42%, and 95.51%, respectively. However, when the temperature is 205 °C, the extraction rate of Ti decreases to 67.26%, while the extraction rates of Al and Mg still remain above 90%. Figure 7 depicts the XRD variations of acid-leached residues at different reaction temperatures. It is evident from the figure that the main acid-leached residue is anhydrite and a small amount of perovskite at temperatures below 190 °C. When the temperature is 190 °C, the mineral of acid-leached residue is all anhydrite. When the temperature is 205 °C, in addition to anhydrite, there is also some TiOSO_4_H_2_O in acid-leached residue. By observing the color of the acid-leached residue at 205 °C, the presence of yellowish particles was found, indicating the presence of iron in the form of oxides in the TBFS. The corresponding iron oxide peaks were not observed in the XRD pattern at 205 °C, indicating that iron ions might have entered the microcrystalline lattice of TiOSO_4_H_2_O or attached to the surface of TiOSO_4_H_2_O in the form of highly dispersed polymer [26]. The reason for the change in the extraction rate of components is the poor reactivity of perovskite, which cannot react effectively with the sulfuric acid solution at lower temperatures, resulting in a relatively low extraction rate of Ti [27]. However, when the temperature is 205 °C, titanium in the form of TiO^2+^ in the solution may be hydrolyzed to generate metatitanic acid, which reacts with sulfuric acid to precipitate TiOSO_4_H_2_O under high temperature and high pressure [28,29], thus reducing the extraction rate of Ti components. In summary, the optimal reaction temperature for acid leaching is 190 °C.

### 2.4. Effect of TBFS Particle Size on Extraction Rates of Ti, Al, and Mg

**Figure 8:**
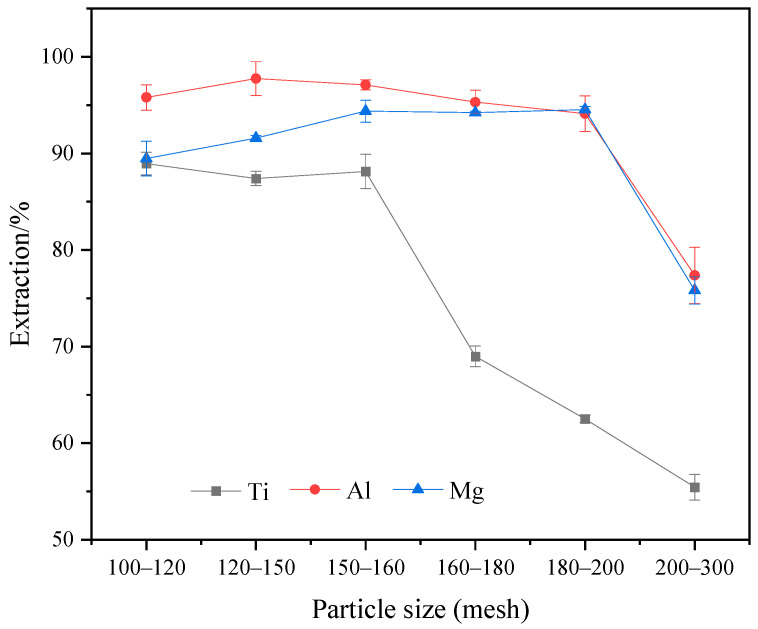
Effect of different TBFS particle sizes on the extraction rates of Ti, Al, and Mg (ωH_2_SO_4_: 55%, temperature: 190 °C, acid–slag: 10 mL/g, time: 2 h).

**Figure 9:**
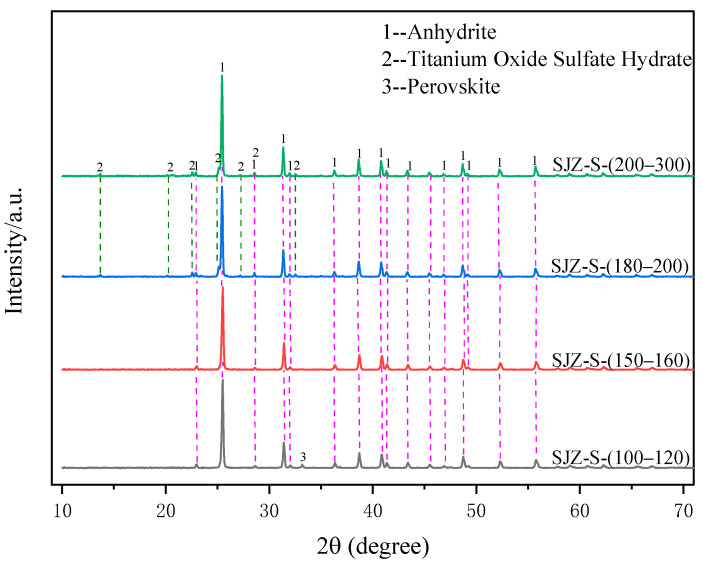
XRD patterns of acid−leached residues under different TBFS particle sizes.

Figure 8 shows the variation in extraction rates of Ti, Al, and Mg components at different material particle sizes. It can be observed from the figure that when the TBFS particle size is larger than 160 mesh, the extraction rate of Ti exhibits a smooth linear trend and remains above 87% as the particle size decreases. However, with the continuous reduction in particle size, the extraction rate of Ti shows a declining trend. When the material particle size is between 100–200 mesh, the extraction rates of Al and Mg remain consistently above 90% with less fluctuations. When the material particle size decreases below 200 mesh, the extraction rates of Al and Mg decrease to 77.38% and 75.82%, respectively. Figure 9 shows the XRD patterns of the acid-leached residues under different TBFS particle sizes. It is evident from the figure that when the material particle size is 100–120 mesh, the main phases in the leaching residue are anhydrite and a small amount of perovskite. When the material particle size decreases to 150–160 mesh, the leaching residue mainly consists of anhydrite. Further reduction in particle size results in the precipitation of TiOSO_4_H_2_O crystals in the acid-leached residue. The reason for the variation in extraction rates of Ti, Al, and Mg components is finer particle size leading to faster reaction rates, thus the decline in Ti extraction rate between 160–200 mesh. This leads to the prolonged presence of TiO^2+^ in the sulfuric acid solution under high-temperature and high-pressure conditions, and the gradual precipitating of TiOSO_4_H_2_O crystals, which reduces the content of Ti^4+^ in the solution, leading to a decrease in the extraction rate of Ti [29]. However, when the TBFS particle size decreases below 200 mesh, the reaction rate is accelerated after the addition of sulfuric acid due to the particle size being too fine, which would result in significant agglomeration that hinders the diffusion of Ti, Al, and Mg components into the solution, thus reducing the overall extraction rates of Ti, Al, and Mg.

Considering energy consumption and extraction rates of Ti, Al, and Mg components, the material particle size of 100–160 mesh was considered more suitable for subsequent experiments.

### 2.5. Effect of Acid–Slag Ratio on the Extraction Rate of Ti, Al, and Mg

**Figure 10:**
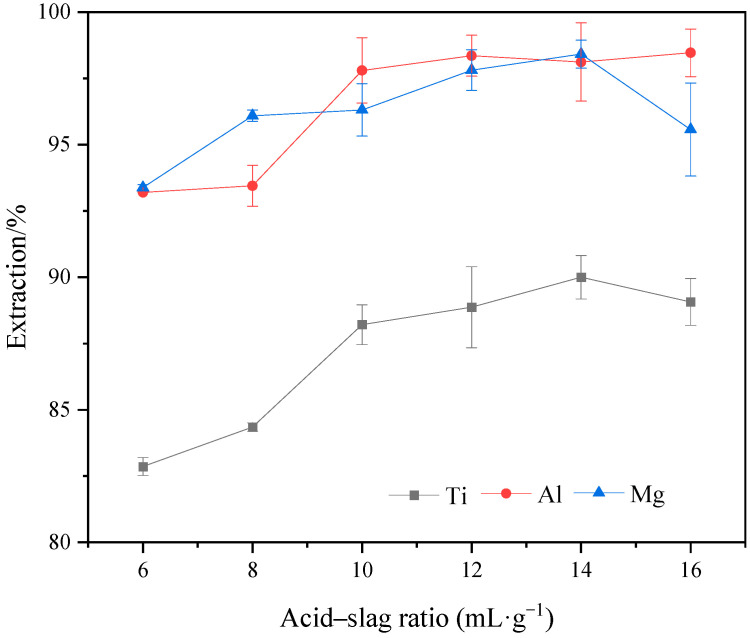
Effect of different acid–slag ratios on the extraction rates of Ti, Al, and Mg (ωH_2_SO_4_: 55%, temperature: 190 °C, time: 2 h, particle size: 100–160 M).

**Figure 11:**
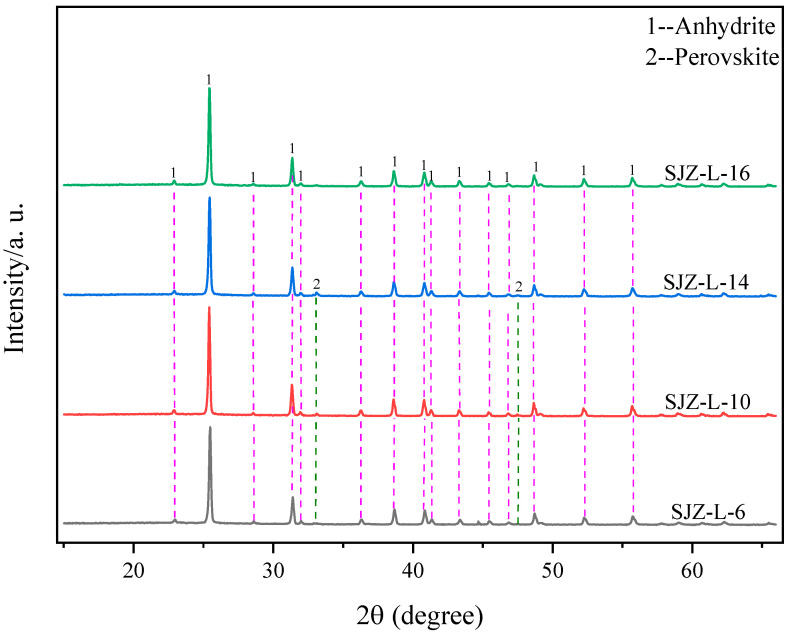
XRD patterns of acid-leached residues under different acid–slag ratios.

Figure 10 shows the variation in Ti, Al, and Mg extraction rates under different acid–slag ratios. It can be observed from the figure that the extraction rates of Ti, Al, and Mg increased with the increase in acid–slag ratio. When the acid–slag ratio reached 10, the extraction rates of Ti, Al, and Mg increased to 88.21%, 97.8%, and 96.31%, respectively. When the acid–slag ratio was greater than 10, the extraction rates of Ti, Al, and Mg fluctuated within a small range without a significant increase. Figure 11 shows the XRD variation in acid-leached residues under different acid–slag ratios. It can be seen from the figure that the main mineral phases of acid-leached residues under different acid–slag ratios are anhydrite and minor amounts of perovskite, the diffraction peak intensities show minimal differences. The analysis of the variation in extraction rates is attributed to several factors. When the acid–slag ratio is less than 10, the presence of excessive reaction residues leads to the formation of clumps during the reaction, which severely hinder the diffusion of reactants to the reaction interface and the diffusion of products into the medium [30]. In addition, the excessive amount of reaction materials causes a gradual decrease in sulfuric acid concentration during the reaction, resulting in incomplete reactions of the remaining reaction materials and reducing the overall extraction rates of Ti, Al, and Mg components. On the contrary, when the acid–slag ratio exceeded 10, the extraction rates of Ti, Al, and Mg were stabilized due to the complete reaction of TBFS [31]. Therefore, the acid–slag ratio of 10 mL/g was considered the optimal value to balance material utilization and acid consumption, and to avoid resource wastage and environmental pollution.

### 2.6. Effect of Reaction Time on the Extraction Rate of Ti, Al, and Mg

**Figure 12:**
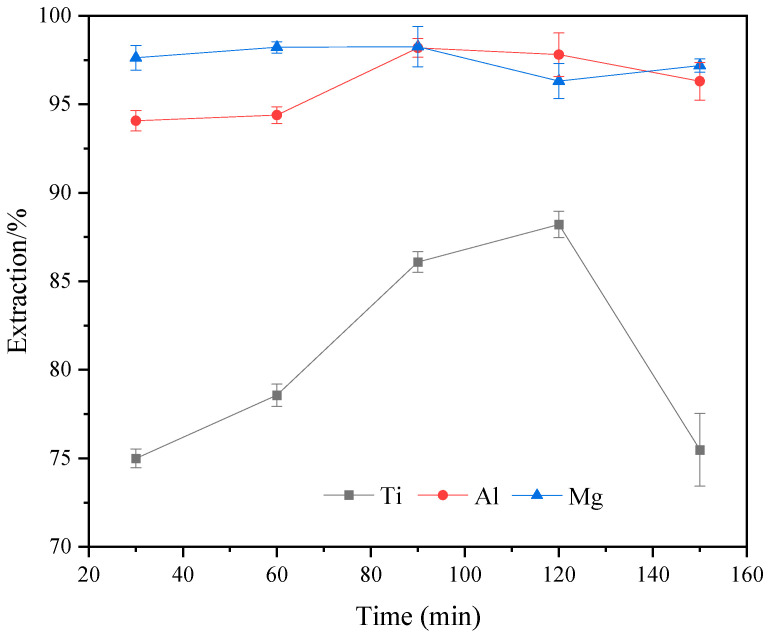
Effect of different reaction times on the extraction rates of Ti, Al, and Mg (ωH_2_SO_4_: 55%, temperature: 190 °C, acid–slag: 10 mL/g, particle size: 100–160 M).

**Figure 13:**
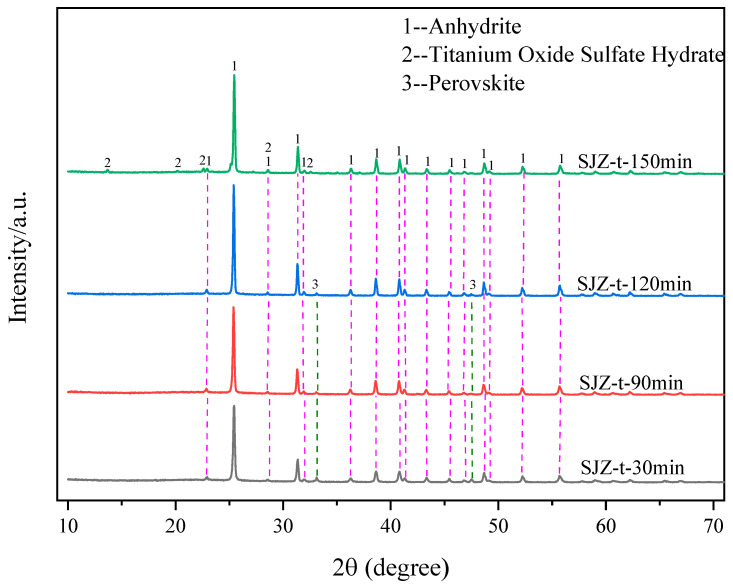
XRD patterns of acid-leached residues under different reaction times.

Figure 12 shows the variation in the extraction rates of Ti, Al, and Mg at different reaction times. With the extension of reaction time, the extraction rate of Al and Mg showed a dynamic trend and remained above 94%. However, the extraction rate of Ti first increased and then decreased with prolonged reaction time. When the reaction time was 120 min, the extraction rate of the Ti component reached the maximum value of 88.21%. Figure 13 shows the XRD variation in acid-leached residues at different reaction times. When the reaction time was less than 120 min, the main phases of the acid-leached residue were anhydrite and a small amount of perovskite. When the reaction time reached 150 min, the acid-leached residues mainly consisted of anhydrite and some precipitation of TiOSO_4_H_2_O crystals. The variation in Ti extraction rate is attributed to the solubility of amorphous Ti component of TBFS within a short duration, whereas the low reactivity of perovskite makes leaching more difficult. Consequently, the leaching rate of perovskite increased and the Ti concentration also increased in the sulfuric acid solution as the reaction time was extended. However, as the reaction time extended to 150 min, titanium ions existing as TiO^2+^ in the sulfuric acid solution precipitated as TiOSO_4_H_2_O crystals, thereby reducing the extraction rate of the Ti component. Considering these factors, the optimal reaction time was determined to be 120 min.

According to the analysis of 2.2–2.6, the optimal extraction rates of Ti, Al, and Mg were found to be 88.21%, 97.8%, and 96.31%, respectively. He et al. [32] extracted valuable components by roasting TBFS with concentrated sulfuric acid and leaching with deionized water. The results show that the extraction rates of Ti, Al, and Mg were 82.85%, 93.16%, and 96.96%, respectively. Comparing the above two methods of extracting valuable components, it was found that the extraction rate of Ti and Al in this study was higher than that of He. Therefore, the leaching method described in this paper is of great significance in improving the extraction rate of valuable components.

### 2.7. FTIR Spectroscopy Analysis

**Figure 14:**
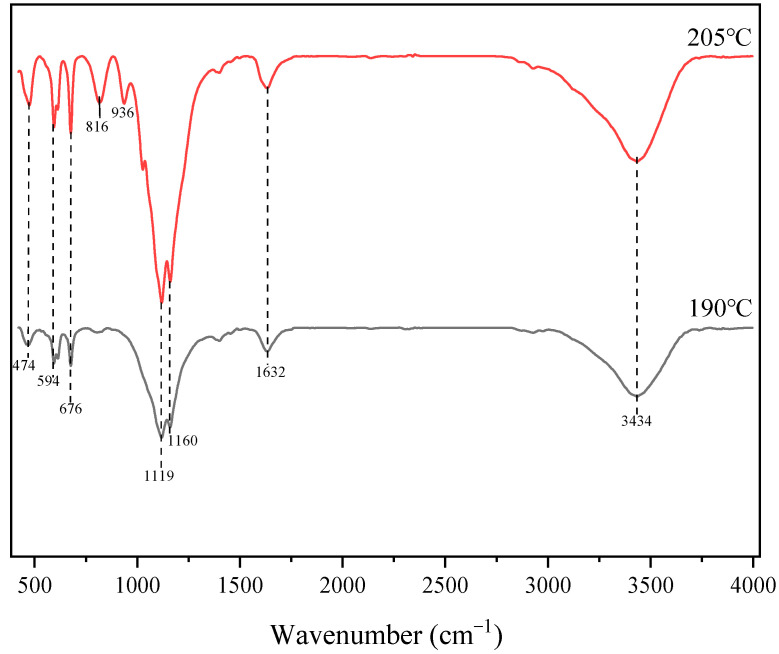
FTIR spectroscopy of the acid-leached residues at 205 °C and 190 °C (ωH_2_SO_4_: 55%, acid–slag: 10 mL/g, time: 2 h, particle size: 100–180 M).

Figure 14 shows FTIR spectroscopy of the acid-leached residues at 205 °C and 190 °C. It can be observed from the figure that the main absorption peaks of the acid-leached residue are around 474 cm^−1^, 594 cm^−1^, 676 cm^−1^, 1119 cm^−1^, 1160 cm^−1^, 1632 cm^−1^, and 3434 cm^−1^ at 90 °C. The main absorption peaks of the acid-leached residue increased by 816 cm^−1^ and 936 cm^−1^ at 205 °C. The wide peak at 3434 cm^−1^ is the stretching vibration peak of the O-H bond, which is related to the surface hydroxyl group and adsorbed water [33]. The absorption peak of 1632 cm^−1^ is produced by the bending vibration of the H-O-H bond [34]. The absorption peaks of 594 cm^−1^, 676 cm^−1^, 1119 cm^−1^, and 1160 cm^−1^ are characteristic peaks of anhydrite [35,36]. The absorption peaks of 816 cm^−1^ and 936 cm^−1^ are assigned to the vibrations of Ti–O or Ti–O–Ti structural groups [37,38]. The absorption peak of 474 cm^−1^ is produced by the vibration of the Si-O bond [39]. Therefore, the results of FTIR spectra are consistent with the XRD analysis in Figure 7. The phase of the acid-leached residue mainly consists of anhydrite and TiOSO_4_H_2_O at 205 °C. And the phase of the acid-leached residue is mainly anhydrite at 190 °C. Therefore, a high temperature for a certain time is unfavorable to the extraction rate of titanium.

### 2.8. The Benefits of Economy and Environment

In this paper, the process leaches Ti, Mg, and Al in TBFS as the target components, and extracts them to prepare corresponding chemical products. The treated residue mainly contains Ca and Si components, which can be used as building raw materials. Theoretically, a 100% resource treatment of TBFS can be realized. On the one hand, the effective use of TBFS and the sale of related industrial products not only bring additional economic income for enterprises or regions, but also reduce the waste of resources, enhancing the stability and sustainability of economic development, and on the other hand, the pollution risk of water, soil, and atmosphere environment in the open mine of TBFS is reduced.

## 3. Materials and Methods

### 3.1. Experimental Materials

The experimental materials used in this paper were water-quenched high-titanium blast furnace slag obtained from Panzhihua Steel Company (Panzhihua, China), grinding to less than 100 mesh.

Chemical composition analysis of TBFS was tested with X-ray fluorescence spectrometry. The results shown in Table 1 indicate that the major components include CaO, SiO_2_, TiO_2_, Al_2_O_3_, and MgO, with minor amounts of SO_3_, Fe_2_O_3_, K_2_O, and MnO.

**Figure 15:**
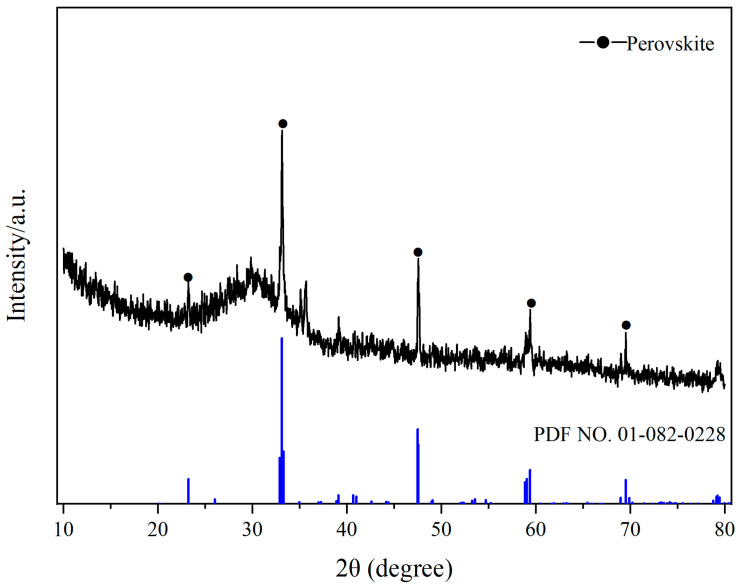
XRD pattern of high-titanium blast furnace slag.

Figure 15 shows the X-ray diffraction (XRD) pattern of TBFS. According to a comparison of standard cards, the diffraction peaks at 23.231°, 33.158°, 47.563°, 59.395°, and 69.522° belong to the characteristic diffraction peak of perovskite (PDF NO. 01-082-0228) [40]. The chemical formula is CaTiO_3_. Additionally, a broad and weak intensity diffraction peak can be observed in the diffraction pattern, indicating the presence of an amorphous phase structure.

### 3.2. Experimental Principles

The valuable components in TBFS include CaO, TiO_2_, Al_2_O_3_, and MgO. Among these, CaO and MgO are basic oxides, while TiO_2_ and Al_2_O_3_ are amphoteric oxides. Therefore, all four valuable components can react with sulfuric acid to form the corresponding sulfate salts. In this process, Ca^2+^ precipitates into the solid phase, while Ti^4+^, Mg^2+^, and Al^3+^ form soluble sulfate salts that enter the liquid phase. The main reaction equations involved in the experimental process are as follows (2)–(7):

(2) $$CaO+H_{2}{SO}_{4}=CaSO_{4}↓+H_{2}O$$

(3) $$TiO_{2}+H_{2}{SO}_{4}=TiOSO_{4}+H_{2}O$$

(4) $${Al}_{2}O_{3}+3H_{2}SO_{4}={Al}_{2}{\left(SO_{4}\right)}_{3}+3H_{2}O$$

(5) $$MgO+H_{2}SO_{4}=MgSO_{4}+H_{2}O$$

(6) $${Fe}_{2}O_{3}+3H_{2}SO_{4}={Fe}_{2}{\left(SO_{4}\right)}_{3}+3H_{2}O$$

(7) $$FeO+H_{2}SO_{4}=FeSO_{4}+H_{2}O$$

### 3.3. Experimental Procedures

In this experiment, TBFS sieved through a 100-mesh screen was used as the raw material. Under conditions of acid leaching at 110 °C, a sulfuric acid concentration of 30% (by mass), an acid–slag ratio of 10, and a leaching time of 4 h, experiments were conducted using both ordinary and pressurized acid leaching methods. For the ordinary condition setting, the conical bottle with TBFS slurry was placed into a magnetic agitator at a certain temperature, and the conical bottle was connected with a condensing reflux device to prevent the water vapor from running out for a period of time, and vacuum filtration was used for liquid–solid separation. For the pressurized condition setting, the reactor with TBFS slurry was placed into an oven at a certain temperature for a period of time, and vacuum filtration was used for liquid–solid separation. The filtration efficiency (F) of the acid-leached slurry was determined for both conditions.

The filtration efficiency (F%) was calculated using Formula (8):

(8) $$F\%=\frac{V_{1}}{V_{0}}\times 100$$

Here, V_0_ represents the initial volume of dilute sulfuric acid added, which is 50 mL, and V_1_ is the volume of the acid-leached solution obtained through vacuum filtration, measured in milliliters (mL).

Different particle sizes of TBFS (100–120 M, 120–150 M, 150–160 M, 160–180 M, 180–200 M, 200–300 M) were mixed with sulfuric acid of varying concentrations (30%, 40%, 50%, 55%, 60%, 70%) at specified acid–slag ratios (6, 8, 10, 12, 14, 16) in reaction kettles, and reacted at different temperatures (100 °C, 115 °C, 130 °C, 145 °C, 160 °C, 175 °C, 190 °C, 205 °C) for different periods of time (30 min, 60 min, 90 min, 120 min, 150 min). After the reactions, vacuum filtration was used for liquid–solid separation. The filter residue was dried at 60 °C for 1 h and analyzed using X-ray diffraction (XRD) to determine the mineral phase composition. The filter residues were marked with SJZ-ω/T/S/L/t. All experiments were carried out in duplicate. The analysis of metal ion content was based on the national standards of the People’s Republic of China. Content of Ti^4+^, Al^3+^ and Mg^2+^ in liquid phase were estimated by titration.

The extraction rate (E%) is defined as the amount of metal M extracted to the liquid phase over the total amount of metal ion in the TBFS. It can be defined as Equation (9):

(9) $$E\%=\frac{M_{1}}{M_{0}}\times 100$$

Here, M_1_ is the metal content in the acid-leached liquid phase. M_0_ is the initial metal content in the TBFS.

### 3.4. Content Analysis Method

#### 3.4.1. Testing Principle of Titanium Content

The tetravalent titanium was reduced to trivalent titanium by aluminum sheet in the carbon dioxide atmosphere. The trivalent titanium solution was titrated by the standard solution of NH_4_Fe (SO_4_)_2_ with NH_4_SCN as an indicator. According to the standard solution of NH_4_Fe (SO_4_)_2_ consumed, the content of titanium in the solution was calculated (GB/T 4701.1-2009) [41]. The main reaction equations involved in the experimental process are as follows (10)–(12):

(10) $$6Ti{\left(SO_{4}\right)}_{2}+2Al=3{Ti}_{2}{\left({SO}_{4}\right)}_{3}+{Al}_{2}{\left({SO}_{4}\right)}_{3}$$

(11) $${Ti}_{2}{\left({SO}_{4}\right)}_{3}+2{NH}_{4}Fe{\left({SO}_{4}\right)}_{2}=2Ti{\left(SO_{4}\right)}_{2}+2Fe{SO}_{4}+{\left({NH}_{4}\right)}_{2}SO_{4}$$

(12) $${NH}_{4}Fe{\left({SO}_{4}\right)}_{2}+3NH_{4}SCN=Fe{\left(SCN\right)}_{3}+2{\left({NH}_{4}\right)}_{2}SO_{4}$$

#### 3.4.2. Testing Principle of Aluminum Content

EDTA was combined with Al^3+^ in aluminum-containing solution in a 1:1 ratio. The EDTA replaced by NaF was titrated by the standard solution of cupric sulfate with PAN as an indicator. The content of aluminum ion in liquid phase was calculated according to the consumption of the standard titration solution of cupric sulfate (GB/T 4701.6-2008) [42]. The main reaction equations involved in the experimental process are as follows (13)–(15):

(13) $${Al}^{3+}+H_{2}Y^{2-}=AlY^{-}+2H^{+}$$

(14) $$AlY^{-}+6NaF+2H^{+}={Na}_{3}AlF_{6}+3{Na}^{+}+H_{2}Y^{2-}$$

(15) $$H_{2}Y^{2-}+{Cu}^{2+}=CuY^{2-}+2H^{+}$$

#### 3.4.3. Testing Principle of Magnesium Content

The interfering elements such as manganese, iron, aluminum, titanium, and vanadium were separated by C_6_H_12_N_4_ and (C_2_H_5_)_2_NCS_2_Na·3H_2_O. The content of calcium was titrated with the standard solution of EDTA in pH ≥ 12 using calcein as an indicator, and the combined content of calcium and magnesium was titrated with the standard titration solution of EDTA in pH = 10 with eriochrome black T as an indicator. The content of magnesium in the solution was calculated according to the combined content of calcium and magnesium and the content of calcium (GB/T 1511-2016) [43].

### 3.5. Sample Characterization

X-ray diffraction (XRD) analysis was conducted to determine the mineral phase composition of the samples using a X’pert Pro X-ray diffractometer (PANalytical B.V., Almelo, The Netherlands). The instrument settings were as follows: copper (Cu) target, tube current of 40 mA, tube voltage of 40 kV, divergence slit (DS) of 0.5°, anti-scatter slit (SS) of 0.04 rad, receiving slit (AAS) of 5.5 mm, scan step size of 0.02°, scan range from 3° to 80° (2θ), and continuous scanning mode.

X-ray fluorescence (XRF) analysis was conducted to determine the chemical composition of the samples using an axios X-ray fluorescence spectrometer (PANalytical B.V., Almelo, The Netherlands). The instrument specifications included a ceramic X-ray tube (Rh target) with a maximum power of 2.4 kW, employing a θ/2θ scanning mode with angle reproducibility better than ±0.0001° and precision of 0.0025°. The detector used was a combination of scintillation and flow gas detectors, capable of analyzing elements ranging from fluorine (F) to uranium (U) (atomic numbers 9 to 92) with a detection range from 0.01% to 100% content.

Fourier transform infrared spectroscopy (FTIR) was used. The test instrument was Frontier FTIR Spectrometer from PerkinElmer of the US. The sample was prepared by a KBr laminating method. The scanning range was 4000~400 cm^−1^, the resolution was 0.4 cm^−1^, and the scanning speed was 0.1681~3.1647 cm/s.

## 4. Conclusions

By comparing acid-leached slurry under ordinary pressure conditions and pressurized conditions, it is found that the acid-leached slurry under ordinary pressure presents a gel-like viscous state, and the acid-leached slurry under pressurized conditions presents an obvious solid–liquid layered state. Furthermore, under ordinary and pressurized conditions, the filtration efficiency of the acid-leached slurry at 10 min was 48% and 78%, respectively.

The optimum process conditions for pressurized leaching were a sulfuric acid concentration of 55%, reaction temperature of 190 °C, TBFS particle size of 100–160 mesh, acid–slag ratio of 10 mL/g, and reaction time of 120 min. The extraction rates of Ti, Al, and Mg components were more than 88.21%, 97.8%, and 96.31%, respectively.

The XRD results of all acid-leached residues revealed that the main component was anhydrite according to a single factor experiment. XRD and FTIR showed that the titanium oxide sulfate hydrate crystals were produced in the acid-leached residues when the reaction temperature exceeded 190 °C, thereby reducing the extraction rate of the Ti component. Additionally, the XRD pattern showed that the titanium oxide sulfate hydrate crystals would also be produced when the temperature was maintained at 190 °C and the reaction time was extended to 150 min. In summary, it was crucial for maintaining a certain reaction temperature and time to improve the extraction rate of titanium.

According to the improved method in this paper, the formation of diaqueous calcium sulfate colloid was avoided, the extraction rate of valuable components was increased, the filterability of the acid leaching system was improved, and the process flow of the sulfuric acid treatment of TBFS was simplified by reducing the process step of water leaching, which provided a new and improved idea for the high-value utilization of TBFS in the future. In future research, Ti in the leaching solution could be obtained by hydrolytic and forging. Mg-Al-Ti-layered double hydroxides were synthesized using Ti, Mg, and Al in the leaching solution as raw materials, which on the one hand could reduce the utilization cost of Ti, Mg, and Al, and on the other hand, by introducing the Ti element, Mg-Al-layered double hydroxides could have traditional adsorption properties, and could also produce photocatalytic properties, so as to realize the comprehensive utilization of high-value Ti, Mg, and Al elements.

## Figures and Tables

**Table 1:** Chemical composition of TBFS (mass fraction, %).

**Component**	CaO	SiO_2_	TiO_2_	Al_2_O_3_	MgO	SO_3_	Fe_2_O_3_
**Content**	28.08	26.74	19.65	13.86	7.64	1.05	0.79
**Component**	K_2_O	MnO	Na_2_O	F	BaO	SrO	ZrO_2_
**Content**	0.72	0.64	0.53	0.17	0.07	0.04	0.02

## Data Availability

The original contributions presented in the study are included in the article; further inquiries can be directed to the corresponding authors.
